# Aeolian Prokaryotic Communities of the Global Dust Belt Over the Red Sea

**DOI:** 10.3389/fmicb.2020.538476

**Published:** 2020-11-12

**Authors:** Nojood A. Aalismail, Rubén Díaz-Rúa, David K. Ngugi, Michael Cusack, Carlos M. Duarte

**Affiliations:** ^1^Red Sea Research Center and Computational Bioscience Research Center, King Abdullah University of Science and Technology, Thuwal, Saudi Arabia; ^2^Leibniz Institute DSMZ – German Collection of Microorganisms and Cell Cultures GmbH, Braunschweig, Germany

**Keywords:** Red Sea atmosphere, airborne prokaryotes, bioaerosols, global dust belt, aeromicrobiology

## Abstract

Aeolian prokaryotic communities (APC) are important components of bioaerosols that are transported freely or attached to dust particles suspended in the atmosphere. Terrestrial and marine ecosystems are known to release and receive significant prokaryote loads into and from the surrounded atmospheric air. However, compared to terrestrial systems, there is a lack of microbial characterization of atmospheric dust over marine systems, such as the Red Sea, which receives significant terrestrial dust loads and is centrally located within the Global Dust Belt. Prokaryotic communities are likely to be particularly important in the Global Dust Belt, the area between the west coast of North Africa and Central Asia that supports the highest dust fluxes on the planet. Here we characterize the diversity and richness of the APC over the Red Sea ecosystem, the only sea fully contained within the Global Dust Belt. MiSeq sequencing was used to target 16S ribosomal DNA of two hundred and forty aeolian dust samples. These samples were collected at ∼7.5 m high above the sea level at coastal and offshore sampling sites over a 2-year period (2015–2017). The sequencing outcomes revealed that the APC in the atmospheric dust is dominated by Proteobacteria (42.69%), Firmicutes (41.11%), Actinobacteria, (7.69%), and Bacteroidetes (3.49%). The dust-associated prokaryotes were transported from different geographical sources and found to be more diverse than prokaryotic communities of the Red Sea surface water. Marine and soil originated prokaryotes were detected in APC. Hence, depending on the season, these groups may have traveled from other distant sources during storm events in the Red Sea region, where the APC structure is influenced by the origin and the concentration of aeolian dust particles. Accordingly, further studies of the impact of atmospheric organic aerosols on the recipient environments are required.

## Introduction

Atmospheric transport plays a major role in the dispersal of microorganisms (Foroutan et al., 2017; Mayol et al., 2017), including the Aeolian Prokaryotic Community (APC). The APC is a complex community that may include pathogens and organisms containing toxins harmful to plants and animals, including humans (Yamaguchi et al., 2012b). Due to their small size (around 1 μm), APC remains in the atmospheric aerosols for long periods crossing long distance (Després et al., 2012; Mayol et al., 2017), particularly when the organisms are inherently adapted for long-range transport by having, for instance, repair mechanisms that protect them against UV-induced DNA damage, or the capacity to remain airborne (Aalismail et al., 2019).

Wind-blown dust is one of the main components of atmospheric aerosols (Chen et al., 2017), particularly in the so-called “global dust belt,” which extends from the west coast of North Africa, over the Middle East, Central and South Asia, to China (Prospero et al., 2002). The “global dust belt” contributes up to 70% of the current global annual dust emission flux (Jickells et al., 2005). The Red Sea, the only body of seawater fully contained within the global dust belt region, receives an estimated 6 Mt of dust annually (Prakash et al., 2015) as a consequence of its location between North Africa and the Arabian Peninsula. The intense and periodic dust cover originating in the drylands (Schroeder, 1985) implies that the Red Sea is a major sink for dust and associated aeolian biota suspended within the global dust belt (Prakash et al., 2015). However, it remains unclear what influence – if any – the APC has on the pelagic microbiome of the Red Sea.

Dust storm formation has intensified due to global warming effects, as a result of the increasing energy transferred from a warmer ocean to the atmosphere (Middleton et al., 2019). The Red Sea is also warming three-times faster than the global ocean warming rate (Chaidez et al., 2017), which could play an important role in the activity of dust storm events. The Red Sea is extremely nutrient deficient, particularly in the central region (Raitsos et al., 2013). Thus, dust deposition has been suggested to play a significant role in delivering nutrients, possibly enhancing biological productivity in the Red Sea (Osipov and Stenchikov, 2018). On the other hand, the global dust belt over the Red Sea carries significant loads of microbes (Yahya et al., 2019), suggested to include communities with an aeolian lifestyle (Aalismail et al., 2019). Hence, there is a need to better understand the community structure and possible sources of the APC over the Red Sea relative to pelagic planktonic prokaryotic communities in Red Sea waters. While several studies have employed a variety of sampling methods to chemically and physically characterize aeolian dust over the Red Sea, APC over the Red Sea has received limited attention. Relevant studies include the quantification of the total loads and deposition rates of virus and bacterial cells (Yahya et al., 2019), the diazotrophic community in the APC (Foster et al., 2009; Rahav et al., 2018), the impacts of bioaerosols on the microbial diversity, and primary and bacterial production rates of surface Red Sea water (Mescioglu et al., 2019b) in the northern part of the Gulf of Aqaba. Therefore, the diversity of APC over much of the Red Sea remains insufficiently characterized.

Here we report the community structure of the APC over the Red Sea and compare it to the prokaryotic community in surface waters. To this end, we carried out metabarcoding sequencing of 16S rRNA gene Illumin, MiSeq sequencer. Dust samples and additional surface seawater samples, collected for 2 years on the coastal and offshore regions of the Red Sea. Specifically, we use these data sets to address the following four questions: (1) Do Red Sea surface waters and the overlaying atmosphere share similar prokaryotic communities? (2) Based on the atmospheric transport history of sampled air, what is the influence of prokaryotes long-range transport on communities’ diversity? (3) Does the APC community structure change seasonally? and (4) Is APC diversity related to the concentration of dust and the dust-associated trace element concentrations?

Accordingly, we hypothesize that Red Sea atmospheric conditions may have some effects on the structure of APC during winter when sand storms are more frequent, which may have an impact on the surface water prokaryotic communities.

## Materials and Methods

### Samples Collection

As described in our previous study (Aalismail et al., 2019), regular sampling of Total Suspended Particulates (TSP) was performed using automatic sequential high-volume samplers (MCV-CAV), equipped with TSP cut off inlets at a flow rate of 20 m^3^ hr^–1^ over periods of 24 h to 1 week. Air was sampled through the inlet utilizing an in-built pump. The ambient air was filtered to collect the suspended particles on quartz fiber filters (WhatmanTM 1810-150 Acid Treated TCLP Filter for EPA Method 1311 with Low Metals, diameter: 15 cm, pore size: 0.6–0.8 μm) until the filter was clogged, which involved sampling periods ranging from 24 h, during dust storms, to 1 week, when dust loads were lowest. The high-volume sampler on board the research vessel was mounted on the top deck at an elevation of ∼7.5 m above sea level and equipped with a weather vane, which would switch off the pump and thus cease sampling immediately whenever the sampler was downwind of the ship’s exhaust. The mass concentration of TSP was determined by weighing the filters before and after sampling and expressed as μg m^–1^. Aeolian dust samples were collected from different backward air trajectories (Supplementary Figure S1) at two locations, the coastal and offshore regions of the Red Sea (Supplementary Figure S2) from September 2015 to December 2017 (Supplementary Table S1).

Surface water samples were collected every 2 weeks from June 2016 to November 2016 at the coastal site of the Red Sea (KAUST). Water samples were collected in 100 mL quartz bottles and filtered through 0.22 μm membrane filters (Millipore). Individual filters were stored in 15 mL tubes. All sample filters were immediately frozen at −20°C. We used different filters for air and water samples because the airborne dust particles, where the cells are attached, are large enough [∼ > 2.1 μm (Li et al., 2011)] to be collected on filters with a pore size (0.6–0.8 μm) since the aim was to collect dust-associated cells but sampling of the microorganisms from water requires smaller pore size filters (0.22 μm) as most planktonic cells are free and not attached to large particles, such as dust. The ambient air was filtered to collect the suspended particles on quartz fiber filters (WhatmanTM 1810-150 Acid Treated TCLP Filter for EPA Method 1311 with Low Metals, diameter: 15 cm, pore size: 0.6–0.8 μm) until the filter was clogged, which involved sampling periods ranging from 24 h, during dust storms, to 1 week, when dust loads were lowest. Hence, the sampling strategy was standardized for dust loads, rather than time. However, we also test whether sampling time affected microbial diversity and richness in the dust samples.

In detail, aeolian dust time-series sampling was done over 2 years. Due to environmental reasons, dust samples were collected until the filter was clogged, which involved sampling periods ranging from 24 h, during dust storms, to 1 week, when dust loads were lowest. Two-hundreds forty dust filters were collected from two high-volume dust collectors at two sites (onshore and offshore). All dust samples with ten surface water samples were sequenced. However, after subsampling to 5127 sequences per sample, one hundred and three dust samples were excluded because of having less than 5127 sequences, since some samples have strong inhibition. Besides the subsampling and keeping the sampling with 5,127 sequences per sample, the inclusion of randomly sampled sweater samples was aimed at giving a qualitative picture of the community assembly and composition relative to the dust samples. To clarify, surface water samples were used for location sampling comparison only, therefore, it was not required to have a seasonal sampling. Already we know that the ensemble of pelagic communities in the sampled region is consistently dominated by the same taxa – that is, SAR11 and Cyanobacteria (Rodriguez-Valera et al., 2012; Pearman et al., 2017). The offshore dust samples were collected during research cruises, which require a research vessel and cannot, therefore, be sustained in parallel over 2 years, thereby resulting in the uneven distribution of sample sizes.

### Backward Trajectory Calculation

The trajectories of air masses arriving at the sampling location above the Red Sea were calculated by Hybrid Single-Particle Lagrangian Integrated Trajectory HYSPLIT model from NOAA (available online at https://ready.arl.noaa.gov/) (Cohen et al., 2015). Global Data Assimilation System (GDAS1) was used to obtain the metrological data through the Real-time Environmental Applications and Display System, which offer an archive of meteorological data output graphics. The HYSPLIT backward trajectories at different altitudes of 200 m, 300 m, and 800 m were counted as individual paths in the present study. Backward air mass trajectories were calculated for 120 h counted since the end of sampling with an average sampling time was 3.3 days including 2 days prior to the onset of sampling.

### DNA Extraction

Total DNA was isolated from 250 dust and water filters samples for 16S rRNA amplicon sequencing using the phenol-chloroform extraction protocol, following the same steps reported in Aalismail et al. (2019). A new clean filter was used as the negative control. However, dust loads on control filters were below the detection limit (by weight) and had, accordingly, too little materials to support sequencing [Supplementary Figure S2 from Aalismail et al. (2019)].

### 16S rRNA Gene Amplicon Library Preparation and Sequencing

Two hundred and fifty 16S rRNA gene amplicons were amplified by PCR using the universal primers fused with Illumina adapter overhang nucleotide sequences.

Primer sequences for the 16S rRNA gene were:

Forward primer:

5′TCGTCGGCAGCGTCAGATGTGTATAAGAGACAGC CTACGGGNGGCWGCAG 3′

Reverse primer:

5′GTCTCGTGGGCTCGGAGATGTGTATAAGAGACAG GACTACHVGGGTATCTAATCC 3′,

as reported by Klindworth et al. (2013). The primer pair (0.3 μl, 10 μM) targets a 467-bp region within the hypervariable region V3–V4. Qiagen Taq PCR Master Mix Kit was used, and no-template control was included. A duplicate of each sample was amplified with an initial activation step at 95°C for 5 min, followed by 40 3-steps cycles consisting of 95°C for 30 s, 55°C for 30 s, and 72°C for 30 s; and a final 5 min extension at 72°C. AMPure XP beads (LABPLAN; Naas, Ireland) was used to purify the libraries according to the Illumina 16S metagenomic sequencing library protocol. Amplicons of 16S were targeted to add indexes and Illumina sequencing adapters in the second stage of PCR using Illumina Nextera XT index kits and Qiagen Taq PCR Master Mix Kit. Cycle conditions were 95°C for 3 min, followed by eight cycles of 95°C for 30 s, 55°C for 30 s, and 72°C for 30 s; then a final extent ion of 72°C for 5 min. Libraries were purified according to the Illumina 16S metagenomic sequencing library protocol using AMPure XP beads (LABPLAN; Naas, Ireland).

Libraries were quantified using a Qubit fluorometer, and the purity was checked by gel electrophoresis. The targeted amplicon libraries were mixed in equal concentrations into a pool according to their quantification measurements. The library pool was quantified using Applied Biosystems Power SYBR Green PCR Master Mix and Agilent DNA 1000 Kit (Agilent Technologies) was used to assess the size and purity on Agilent 2100 Bioanalyzer (Agilent Technologies). Six pM of the denatured library pool was spiked with 25% of PhiX Illumina library control. The sequencing run was conducted on the Illumina MiSeq in the Bioscience Core Lab facilities at KAUST.

### Bioinformatics Analysis of Amplicon Library Sequences

A total of 14,680,540 raw sequence reads for all samples in this study were demultiplexed by MiSeq Reporter (v. 2.4.60.8; Illumina, San Diego, CA, United States). Quality was trimmed and filtered using Trimmomatic (Bolger et al., 2014). *Enterobacteria phage phiX174 sensu lato* reads were aligned to the corresponding reference sequences and removed by BBMap (BBMap – Bushnell B)^1^. For error correction, SPAdes (Bankevich et al., 2012) (v3.90) was used. Then, cutadapt v1.13 (Davis et al., 2013) was used to remove adapter sequences from sequencing reads. The PANDAseq (Masella et al., 2012) tool was used to assemble the paired ends of reads, followed by the standard operational procedure for determining operational taxonomic units (OTUs) with MOTHUR (Schloss et al., 2009) pipeline (version 395), including alignment of sequences against the SILVA database (Pruesse et al., 2012; Quast et al., 2013; Yilmaz et al., 2014) and removal of other unwanted sequences (i.e., sequences assigned to chloroplasts, mitochondria, and eukaryotes), as well as chimeras (1,360,272) using VSEARCH (Rognes et al., 2016), and filtering singletons following clustering of OTUs at 97% identity cutoff. From the resulting 7,416,514 sequences (average length of 293 bp), further subsampling of 5,127 sequences per sample was done to facilitate the comparison of community diversity metrics. 103 dust samples were excluded from further analysis because they had less than 5,127 sequences.

### Accession Numbers

All sequence data generated by this study have been submitted to the ENA (European Nucleotide Archive) under the accession number PRJEB36850.

### Statistical Analyses

**FIGURE 1:**
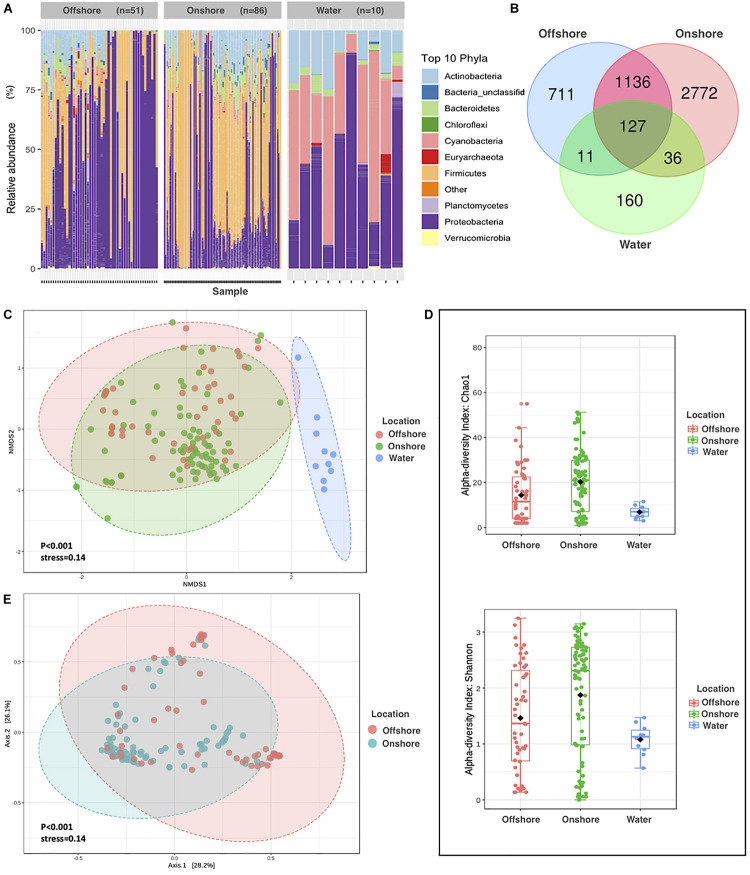
Comparison of Aeolian and Surface-water Prokaryotic Communities in the Red Sea area. **(A)** The relative abundance of APC in onshore air samples, offshore air samples, and surface water samples. **(B)** Venn diagram of unique and common bacterial phyla by sampling locations. **(C)** NMDS on Bray–Curtis distance of prokaryotic for air and water composition. Each data point represents an individual sample, and different colors represent different sampling locations. The distance between points represents the level of difference. Stress lower than 0.2 indicates that the NMDS analysis is reliable. The closer the samples are in the graph, the higher their similarity. **(D)** Alpha diversity measures Chao1 and Shannon for APC in onshore air samples, offshore air samples, and surface water samples. **(E)** Principal coordinate analysis (PCoA) based on the overall structure of APC in onshore and offshore air samples. Each data point represents an individual sample. PCoA was calculated using Bray–Curtis distances with a multivariate t-distribution.

Differences between environmental variables and factors were tested using analysis of variance (Younossi et al., 2018) and *t*-test. A *p*-value of 0.05 was used to determine significance unless noted otherwise. Alpha-diversity calculations and subsequent diversity analyses were performed in R version 1.1.463. The analysis of structural differences between prokaryotic communities (β-diversity) was performed using Bray–Curtis metrics. All statistical analysis and graphs were produced with JMP 14 (SAS Institute Inc., Cary, NC, United States), GraphPad Prism 7.0b (GraphPad Software Inc., San Diego, CA, United States), MicrobiomeAnalyst (Dhariwal et al., 2017), and R software, primarily using the statistical package vegan and the graph package ggplot2^2^ (Wickham, 2016). The numbers of unique and OTUs by sampling locations were visualized using a three-way Venn diagram plot constructed using the online resource of Bioinformatics and Evolutionary Genomics^3^ (Figure 1B).

## Results

We identified a total pool of 5,357 OTUs, distributed among 349 unique prokaryotic phyla in the APC over the Red Sea, where Bacteria dominated the communities, accounting, on average, for 99.82% of the amplicon reads and 98.74% of the OTUs found throughout the study. Reads of 16S rRNA genes that were retrieved from offshore air mostly belonged to the following phyla: Proteobacteria (60%), Firmicutes (28%), Actinobacteria (6%), Bacteroidetes (2.5%), Planctomycetes (0.6%), Cyanobacteria (0.58%), Euryarchaeota (0.33%), Verrucomicrobia (0.18%), and Chloroflexi (0.10%), and Deinococcus-Thermus (0.09%) (Figure 1A). In contrast, reads of 16S rRNA genes that were retrieved from onshore air mostly belonged to the following phyla: Firmicutes (50%), Proteobacteria (33%), Actinobacteria (9%), Bacteroidetes (5%), Cyanobacteria (1.1%), Planctomycetes (0.6%), Verrucomicrobia (0.3%), Chloroflexi (0.24%), Deinococcus-Thermus (0.21%), and Gemmatimonadetes (0.12%). Reads of 16S rRNA genes that were retrieved from surface waters (1 m) mostly belonged to the following phyla: Proteobacteria (46%), Cyanobacteria (35%), Actinobacteria (13%), Bacteroidetes (3%), Euryarchaeota (1%), Planctomycetes (0.8%), Verrucomicrobia (0.2%), Deferribacteres (0.1%), Chloroflexi (0.64%), and Tenericutes (0.02%). Hence, OTUs belonging to Firmicutes were highly represented in both offshore and onshore APCs, while Firmicutes were present in very low abundance in the water samples (Figure 1A). Species richness (OTUs at 97% identity threshold) in individual APC samples ranged from 7 to 730, with an average (±SE) of 190 ± 14.3 OTUs per sample based on the Chao1 index (Figure 1D and Supplementary Table S2).

The diversity of prokaryotic OTUs differed significantly among sampling locations (ANOVA, *P* = 0.001), which were higher in the aeolian samples collected offshore and onshore than in the Red Sea surface seawater samples, where sampling locations explain 14% of the variability. Moreover, there was higher prokaryotic diversity in the air than in the surface water samples (Figure 1D). The number of OTUs shared by the three sampling locations (onshore air, offshore air, and surface water) was only 127 OTUs, whereas 1,136 OTUs were shared between onshore air and offshore air samples (Figure 1B). These results confirm that the airborne and seawater prokaryote communities are fundamentally different. Unexpectedly, the 2,772 OTUs were unique to onshore air samples, meaning they did not match samples from the other sampling locations, whereas 711 and 160 OTUs were unique to offshore air and surface water samples, respectively. We also observed significant clustering (ANOVA, *P* < 0.001) of APCs between onshore and offshore air samples, exclusive of surface water prokaryotic communities (Figure 1C) based on NMDS of Bray–Curtis distances among samples. By performing ANOVA with Bray–Curtis distance, principal coordinates analysis (PCoA) showing the community structure of aeolian prokaryotes differed significantly between onshore and offshore air (*P* < 0.001) (Figure 1E).

**FIGURE 2:**
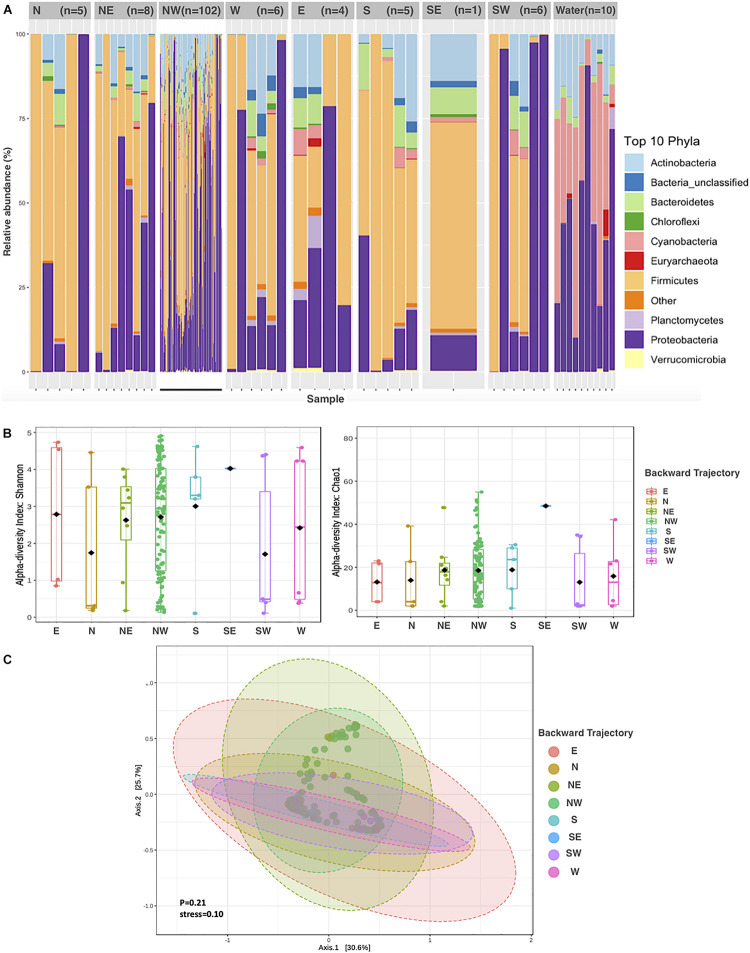
Aeolian Prokaryotic Communities over the Red Sea per air backward trajectories. **(A)** The relative abundance of prokaryotes in the nine sources. **(B)** Alpha diversity measures Chao1 and Shannon for APC in eight air backward trajectories. **(C)** Principal coordinate analysis (PCoA) based on the overall structure of APC in eight air backward trajectories. Each data point represents an individual sample. PCoA was calculated using Bray–Curtis distances with a multivariate t-distribution.

Most of the dust samples (74%) originated from air masses from the northwest (NW, Supplementary Figure S1), the prevailing wind direction in the Red Sea. The contribution of different phyla to APCs suspended in air masses sampled from different trajectories was relatively conserved among air masses, with a dominance of Proteobacteria, Firmicutes, and Actinobacteria (Figure 2A). Though no significant differences in the community composition between APCs were apparent (Figure 2C, ANOVA, *P* = 0.21), the average alpha diversity, however, differed depending on the air mass source, which accounted for 14% of the variability in alpha diversity (*P* = 0.001). The richest and most diverse samples originated from northwestern geographical sources (Figure 2B).

**FIGURE 3:**
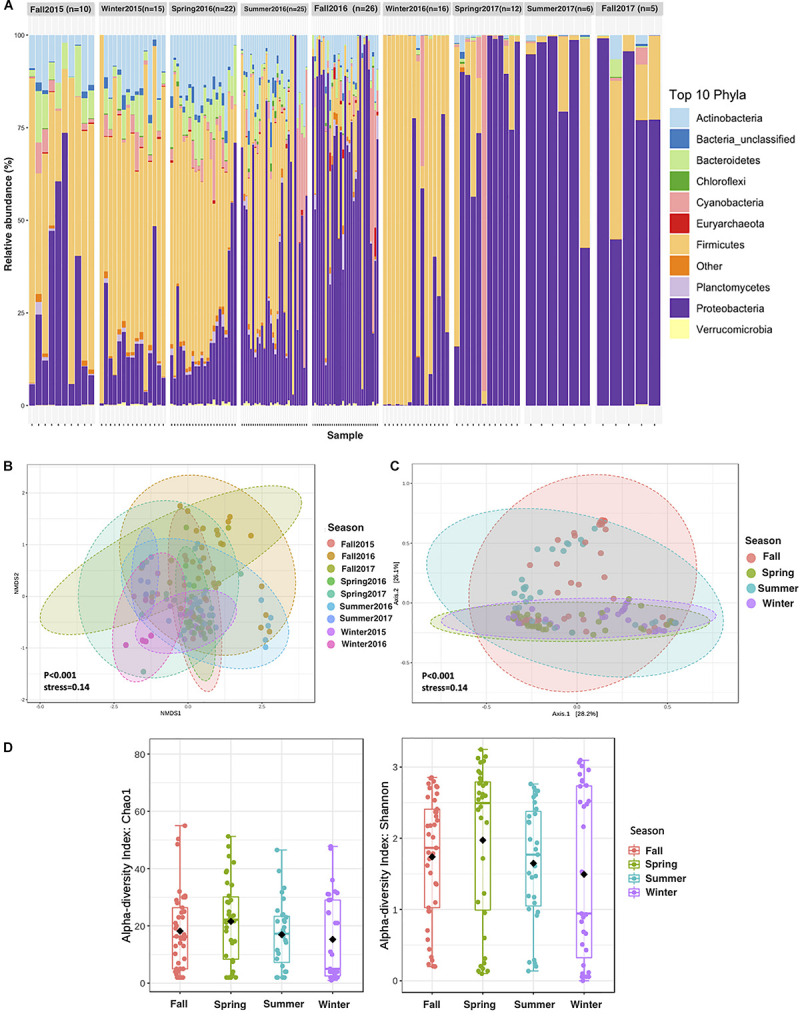
Aeolian Prokaryotic Communities over the Red Sea per sampling season. **(A)** The relative abundance of APC during 2 years of sampling. **(B)** NMDS on Bray–Curtis distance of prokaryotic for air composition during 2 years of sampling. Each data point represents an individual sample. **(C)** Principal coordinate analysis (PCoA) based on the overall structure of APC in the four seasons regardless of the sampling years. Each data point represents an individual sample. PCoA was calculated using Bray–Curtis distances with a multivariate t-distribution. **(D)** Alpha diversity measures Chao1 and Shannon for APC in seasonality.

**FIGURE 4:**
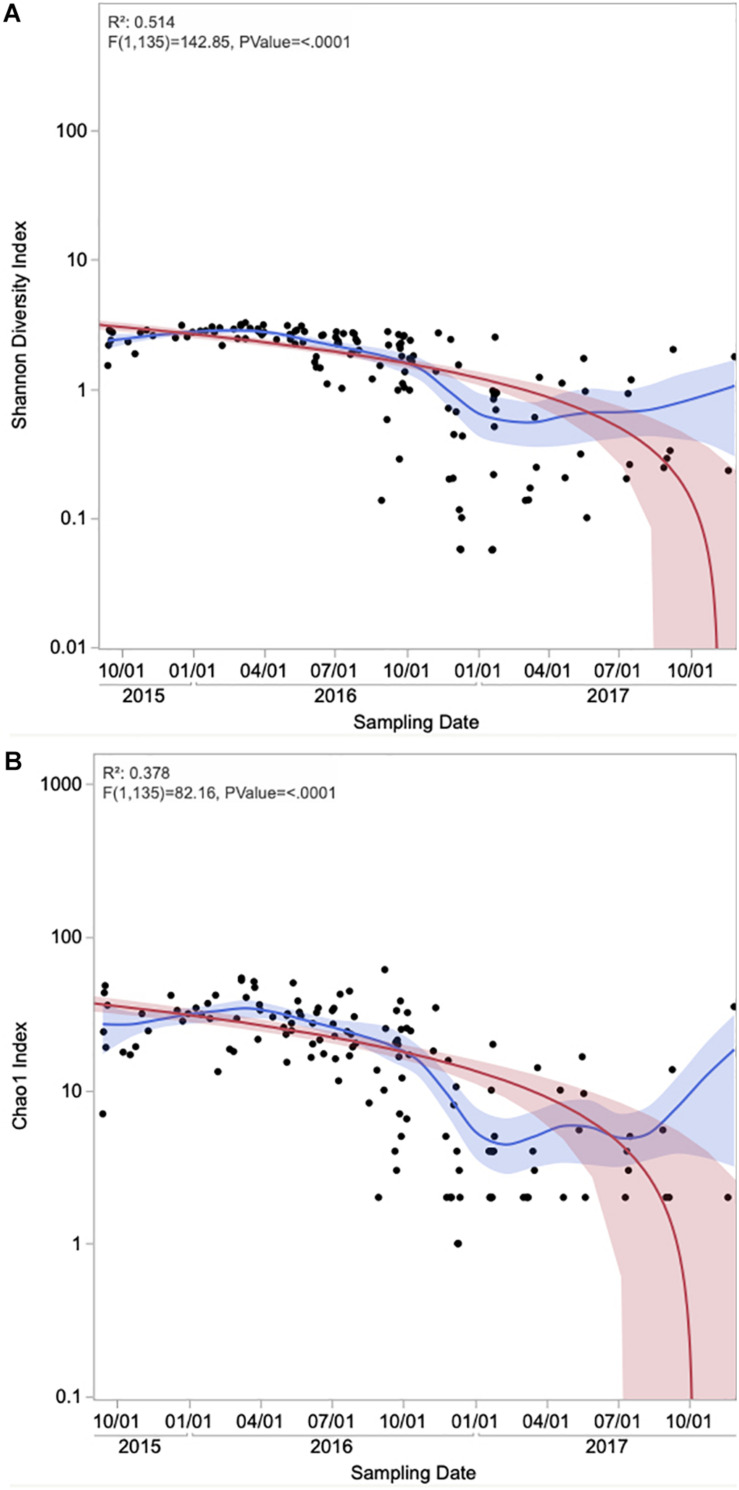
Time series of alpha diversity measures **(A)** Shannon and **(B)** Chao1 of the onshore and offshore atmospheric air above the Red Sea. Each data point represents an individual sample. The Red line represents the regression line and the red band around it represents the 95% confidence for the regression line. The blue line represents the mean values and the blue shaded area is the associated 95% confidence interval.

The contribution of different phyla to APCs sampled in different seasons showed contrasting community structure, with those sampled in fall and summer dominated by Proteobacteria and Actinobacteria. In contrast, those sampled in spring and winter were dominated by Firmicutes (Figure 3A). The average alpha diversity of the APCs differed among seasons, which explained 33% of the variance in community structure (Figures 3B,C, ANOVA, *P* = 0.001). The richest and most diverse samples were those sampled in 2016, with diversity being lowest in summer and highest during spring and winter (Figures 3D, 4). Richness and diversity were significantly different in the sampling seasons (Figure 4).

We used Analysis of Variance, with Bonferroni multiple comparison test correction, to assess the influence of environmental factors on the differences of the dominated phyla relative abundances. The results revealed that relative abundances of Actinobacteria, Bacteria_unclassified, Bacteroidetes, Candidate_division_TM7, Cyanobacteria, Euryarchaeota, Firmicutes, and Proteobacteria differed significantly between sampling locations as shown in (Supplementary Figure S3A). There are strong significant differences (ANOVA, *P* ≤ 0.001) in the relative abundance of Cyanobacteria in (offshore air vs. water and onshore air vs. water), Firmicutes in (offshore air vs. onshore air and onshore air vs. water), and Proteobacteria in (offshore air vs. onshore air). Supplementary Figure S3B indicates that seasonal factors show a strong significant (ANOVA, *P* ≤ 0.001) impact on the differences between relative abundances of Firmicutes in (Fall vs. Winter, Spring vs. Winter, and Summer vs. Winter) and Proteobacteria in (Fall vs. Winter). However, only variances in the relative abundance of unclassified bacteria have been highly significantly influenced by the backward air trajectories (ANOVA, *P* ≤ 0.001) in (NE vs. W, NW vs. W, and S vs. W) (Supplementary Figure S3C).

**FIGURE 5:**
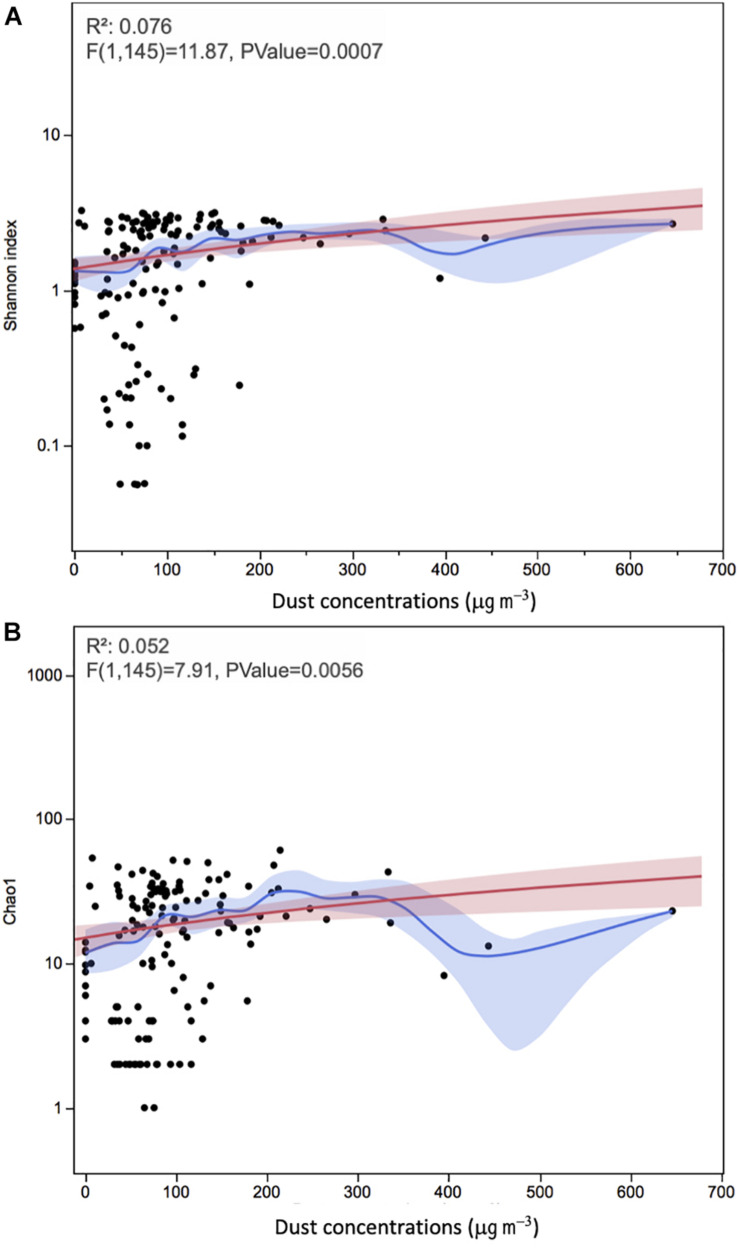
The relationships between alpha diversity measures **(A)** Shannon and **(B)** Chao1 and dust concentrations in onshore and offshore atmospheric air sampling locations above the Red Sea. Each data point represents an individual sample. The red line represents the regression line and the red band around it represents the 95% confidence for the regression line. The blue line represents the mean values and the blue shaded area is the associated 95% confidence interval.

Using ANOVA, we analyzed the linear regression model between dust concentrations and diversity indices (Figure 5). The correlations between dust loads and the APC’s diversity and richness were significant using Shannon and Chao1 indices (*P* = 0.0007 and 0.0056, respectively), but with very low correlation (*R*^2^ = 0.076 and 0.052, respectively). Kruskal–Wallis test showed no significant effect of sampling duration on the microbial diversity and richness of dust samples (*P* > 0.10).

Airborne dust particles also typically have a complex chemical composition, which is known to impact the airborne prokaryotic structure (Innocente et al., 2017). To understand the potential influence of trace element concentrations on the bacterial communities in the Red Sea atmosphere, we undertook principal component analysis (PCA). Measurements of trace elements were obtained from (Cusack et al., unpublished), which uses the same airborne dust filters as those in this study. Supplementary Figure S4A represents the results of PCA at the phylum level of the correlation between trace elements and the variation of APC alpha diversity and relative abundances of the top to abundant phyla in the two air masses sampling sites (onshore and offshore). The horizontal axis in (Supplementary Figure S4A) (PC1) explains 44.2% of the total variance in the data and the vertical axis (PC2) represents an additional 12.5% of the variance. The analysis of the biplot indicates that most of the phyla abundances are not correlated with the trace elements contained in the onshore atmosphere, except the Proteobacteria and Firmicutes. The APC alpha diversity depicted a positive correlation only with the molybdenum. For the offshore sampling site (Supplementary Figure S4B), the first PC explained 44.8% and the second axis explained 18.2% of the total variance. In this PCA biplot, Proteobacteria and Verrucomicrobia are the only two phyla showing a positive correlation with the trace element concentrations.

## Discussion

In-depth analysis of dust-associated prokaryotes in this study provides an insight into the APCs over the Red Sea and reveals a high degree of diversity of the analyzed aeolian dust, consistent with reports of APCs over the Mediterranean Sea (Federici et al., 2018; Mescioglu et al., 2019a) and elsewhere (Hua et al., 2007; Jeon et al., 2011; Davis et al., 2013; Cáliz et al., 2018; Mescioglu et al., 2019b). The APC composition varied over time but was dominated by phylotypes belonging to Proteobacteria, Firmicutes, Actinobacteria, and Bacteroidetes, which are known to include several pathogenic species (Yamaguchi et al., 2012a; Morimoto et al., 2017). These bacterial members are typically generated from soil environments (Kenzaka et al., 2010). Remarkably, a high number of unclassified sequences were present in APCs compared to water samples, which indicates the presence of unknown bacteria in the APC that may be derived from soils and specific to this community.

Unique phylotypes were detected in air above the Red Sea. The presence of 53 euryarchaeal sequences belonging to Euryarchaeota in APCs, especially in fall and summer, may be related to the seasonal dynamics of methanogenic Euryarchaeota. Anthropogenic activities such as fertilization of agricultural fields with biogas substrates or manure may be a source of Euryarchaeota, which are emitted to the atmosphere and subsequently transported to the air (Fröhlich-Nowoisky et al., 2014).

Suspension of silt and sand particles in arid soil leads to high loads of aeolian dust in the arid and semi-arid region, which can then be transported over long distances. A significant number of prokaryotes and other biological particles are entrained into the atmosphere during dust resuspension (Jaenicke, 2005; Griffin et al., 2007), where those microbial cells attach to the surface of airborne dust particles (Li et al., 2011), and transport to long distances (Mayol et al., 2017). We found that members of Firmicutes and Bacteroidetes, which are known to form endospores (Bueche et al., 2013) and attach onto coarse particles (Zhang et al., 2007), respectively, and favor the aeolian lifestyle, increased their relative abundances in the air samples but not in the surface water samples (Figure 1A). We determined that the collected dust particles during winter and spring originated from the Global Dust Belt, primarily the Saharan desert and the Arabian Peninsula. These areas provide the Red Sea atmosphere with populations of Firmicutes and Bacteroidetes, which are known to be maintained suspended in the atmosphere for an extended duration (Rosselli et al., 2015). Moreover, dust storms over the coastal regions adjacent to the Red Sea may resuspend terrestrial particles to the atmosphere, further contributing to the high diversity of aeolian microbial communities. After such dust storms, prokaryotic species that are resistant to harsh atmospheric conditions may remain as the dominant members of the aeolian microbiome. The positive, albeit weak, relationship between APC diversity and dust loads show dust storm events in the Red Sea region tend to lead to diverse APC. Dust concentrations in summer were higher than in other seasons, which is consistent with the higher APC diversity in the summer season found here. Surprisingly, the APCs were less diverse in 2017 compared to 2015 and 2016, possibly because storm events tend to be accompanied by rain^4^, compared to dry dust storms in 2015 and 2016 (Figure 3D).

The comparison of prokaryote communities in Red Sea surface waters and dust showed these communities to be very distinct, with different dominant taxa and a very small fraction of OTUs that were found both in water and air. This is consistent with the finding that the dominant communities present in dust suspended over the Red Sea are characteristic of soils and are unable to grow when deposited in seawater. While only about 5% of OTUs found in the aeolian prokaryote communities sampled here were also found in Red Sea seawater, about half of the OTUs found in Red Sea waters, which had much fewer OTUs than APCs, were also found in the atmosphere. These results suggest that the vast majority of OTUs present in APCs are unable to grow in seawater and are most likely soil bacteria. In contrast, half of the OTUs present in seawater can be resuspended and become transient members of the APCs. Indeed, the APC included cyanobacteria, which are the dominant primary producers in the Red Sea, and were likely resuspended from seawater before the air mass reached the onshore or offshore sampling points.

Sampling location was the main source of differences in relative abundances between dominated airborne prokaryotes, followed by sampling seasons and atmospheric transportation history, respectively. This indicates differences in the sources for airborne prokaryote in onshore air, possibly more influence by local sources, and offshore air, as well as between the atmospheric air and the Red Sea water habitats. Whereas, only unclassified bacterial phyla show differences in relative abundances influenced by their origins and transportation history. Accordingly, the Red Sea atmosphere receives unidentified bacterial taxa.

Many studies have confirmed the influence of atmospheric dust-associated trace elements on the airborne prokaryotic community (Yan et al., 2018; Zhai et al., 2018; Romano et al., 2020). However, there was no clear correlation between APC structure and the trace elements contained in the onshore and offshore atmosphere over the Red Sea as shown in the PCA biplots.

In summary, our results show that a diverse array of APCs are present in the atmospheric air over the Red Sea, where multiple factors influenced the APC composition. We observed high percentages of marine and soil prokaryotes in the air, indicating that there is a significant exchange of microbes between the sea surface, terrestrial environments, and the air, even during non-dust storm conditions. Our results also show, for the first time, the presence of Archaea in the air over the Red Sea (0.18% of reads). Although our results suggest that APCs are unlikely to have a significant influence on Red Sea communities, APCs nevertheless can affect communities in terrestrial ecosystems, including animal and human health (Després et al., 2012). Our results reveal that the APC over the Global Dust Belt is a highly diverse community that provides connectivity across terrestrial ecosystems, but with an inferred small role in affecting marine prokaryote communities.

## Data Availability Statement

Publicly available datasets were analyzed in this study. This data can be found here: All sequence data generated by this study have been submitted to the ENA (European Nucleotide Archive) under the accession number PRJEB36850.

## Author Contributions

CD and NA initiated this study and wrote the manuscript. NA and RD-R designed and tested the extraction approach and extracted and prepared the samples for sequencing. MC collected the samples. NA and DN performed the data analyses. All authors contributed to improving the manuscript, read and approved the final manuscript.

## Conflict of Interest

The authors declare that the research was conducted in the absence of any commercial or financial relationships that could be construed as a potential conflict of interest.

## Footnotes

1 http://sourceforge.net/projects/bbmap/

2 http://www.R-project.org/

3 http://bioinformatics.psb.ugent.be/webtools/Venn/

4 https://phys.org/news/2017-11-nasa-views-severe-storms-western.html
