# Supplementary material for: Aeolian Prokaryotic Communities of the Global Dust Belt Over the Red Sea
Source: Front Microbiol. 2020 Nov 12;11:538476. doi: 10.3389/fmicb.2020.538476 (PMC7688470; doi:10.3389/fmicb.2020.538476)
Supplement: Supplementary file 1 [file Data_Sheet_1.PDF]

Aeolian Prokaryotic Communities of the Global Dust Belt over the Red Sea

Nojood A. Aalismail [1*], Rubén Díaz-Rúa [1], David K. Ngugi [2], Michael Cusack [1], and Carlos M. Duarte [1]

[1]Division of Biological and Environmental Science and Engineering, Red Sea Research Centre and Computational Bioscience Research Center, King Abdullah University of Science and Technology, Thuwal, Saudi Arabia

[2] Leibniz Institute DSMZ — German Collection of Microorganisms and Cell Cultures GmbH Inhoffenstrasse 7B, 38124 Braunschweig, Germany

*** Correspondence:** Nojood A. Aalismail

Nojood.aalismail@kaust.edu.sa

Table S1. Sample metadata. Sample collection data and the backward air masses trajectories at the sampling dates

sample	location	date	season	trajectory	latitude	longitude
KAUST00	Onshore	9/13/15	Fall2015	NW	22.41	39.15
KAUST001	Onshore	9/14/15	Fall2015	NE	22.41	39.15
KAUST002	Onshore	9/15/15	Fall2015	NW	22.41	39.15
KAUST003	Onshore	9/16/15	Fall2015	NW	22.41	39.15
KAUST004	Onshore	9/17/15	Fall2015	NW	22.41	39.15
KAUST005	Onshore	9/19/15	Fall2015	NW	22.41	39.15
KAUST006	Onshore	10/9/15	Fall2015	NW	22.41	39.15
KAUST007	Onshore	10/18/15	Fall2015	NW	22.41	39.15
KAUST008	Onshore	10/25/15	Fall2015	S	22.41	39.15
KAUST009	Onshore	11/2/15	Fall2015	NW	22.41	39.15
KAUST010	Onshore	11/10/15	Fall2015	NW	22.41	39.15
KAUST013	Onshore	12/9/15	Winter2015/2016	NW	22.41	39.15
KAUST014	Onshore	12/17/15	Winter2015/2016	NW	22.41	39.15
KAUST015	Onshore	12/24/15	Winter2015/2016	NW	22.41	39.15
KAUST016	Onshore	12/31/15	Winter2015/2016	NW	22.41	39.15
KAUST017	Onshore	1/10/16	Winter2015/2016	NW	22.41	39.15
KAUST018	Onshore	1/17/16	Winter2015/2016	NE	22.41	39.15
KAUST019	Onshore	1/25/16	Winter2015/2016	NW	22.41	39.15
KAUST020	Onshore	1/28/16	Winter2015/2016	NW	22.41	39.15
KAUST021	Onshore	2/4/16	Winter2015/2016	NW	22.41	39.15
KAUST022	Onshore	2/7/16	Winter2015/2016	S	22.41	39.15
KAUST023	Onshore	3/2/16	Winter2015/2016	NW	22.41	39.15
KAUST024	Onshore	3/2/16	Spring2016	W	22.41	39.15
KAUST025	Onshore	3/8/16	Spring2016	NW	22.41	39.15
KAUST026	Onshore	3/14/16	Spring2016	N	22.41	39.15
KAUST027	Onshore	3/24/16	Spring2016	NW	22.41	39.15
KAUST028	Onshore	4/1/16	Spring2016	NW	22.41	39.15
KAUST029	Onshore	4/5/16	Spring2016	N	22.41	39.15
KAUST030	Onshore	4/17/16	Spring2016	NW	22.41	39.15
KAUST032	Onshore	5/1/16	Spring2016	SW	22.41	39.15
KAUST034	Onshore	5/4/16	Spring2016	SW	22.41	39.15
KAUST035	Onshore	5/5/16	Spring2016	NW	22.41	39.15
KAUST036	Onshore	5/6/16	Spring2016	S	22.41	39.15
KAUST037	Onshore	5/11/16	Spring2016	NW	22.41	39.15
KAUST038	Onshore	5/13/16	Spring2016	NW	22.41	39.15
KAUST039	Onshore	5/19/16	Spring2016	NW	22.41	39.15
KAUST040	Onshore	5/21/16	Spring2016	NW	22.41	39.15
KAUST041	Onshore	5/23/16	Spring2016	NW	22.41	39.15
KAUST043	Onshore	6/5/16	Summer2016	NE	22.41	39.15
KAUST044	Onshore	6/7/16	Summer2016	NE	22.41	39.15

KAUST045	Onshore	6/12/16	Summer2016	NW	22.41	39.15
KAUST046	Onshore	6/14/16	Summer2016	NW	22.41	39.15
KAUST047	Onshore	6/16/16	Summer2016	NW	22.41	39.15
KAUST048	Onshore	6/21/16	Summer2016	NW	22.41	39.15
KAUST052	Onshore	7/3/16	Summer2016	NW	22.41	39.15
KAUST053	Onshore	7/5/16	Summer2016	NW	22.41	39.15
KAUST054	Onshore	7/2/16	Summer2016	NW	22.41	39.15
KAUST055	Onshore	7/4/16	Summer2016	NW	22.41	39.15
KAUST056	Onshore	7/6/16	Summer2016	NW	22.41	39.15
KAUST058	Onshore	7/10/16	Summer2016	NW	22.41	39.15
KAUST059	Onshore	7/12/16	Summer2016	NW	22.41	39.15
KAUST060	Onshore	7/26/16	Summer2016	W	22.31	39
KAUST061	Onshore	7/28/16	Summer2016	NW	22.41	39.15
KAUST062	Onshore	7/30/16	Summer2016	W	22.41	39.15
KAUST063	Onshore	8/1/16	Summer2016	NW	22.41	39.15
KAUST071	Onshore	9/8/16	Fall2016	NW	22.41	39.15
KAUST077	Onshore	9/20/16	Fall2016	NW	22.41	39.15
KAUST078	Onshore	9/22/16	Fall2016	NW	22.41	39.15
KAUST080	Onshore	9/26/16	Fall2016	NW	22.41	39.15
KAUST082	Onshore	10/5/16	Fall2016	NW	22.41	39.15
KAUST091	Onshore	11/11/16	Fall2016	SE	22.41	39.15
KAUST095	Onshore	12/4/16	Winter2016/2017	NW	22.41	39.15
KAUST098	Onshore	12/7/16	Winter2015/2016	NW	22.41	39.15
KAUST099	Onshore	12/8/16	Winter2016/2017	N	22.41	39.15
KAUST100	Onshore	12/9/16	Winter2015/2016	NE	22.41	39.15
KAUST101	Onshore	12/10/16	Winter2016/2017	N	22.41	39.15
KAUST102	Onshore	12/11/16	Winter2016/2017	NW	22.41	39.15
KAUST103	Onshore	12/12/16	Winter2016/2017	NW	22.41	39.15
KAUST107	Onshore	1/18/17	Winter2016/2017	S	22.41	39.15
KAUST108	Onshore	1/19/17	Winter2016/2017	SW	22.41	39.15
KAUST109	Onshore	1/20/17	Winter2016/2017	NW	22.41	39.15
KAUST110	Onshore	1/21/17	Winter2016/2017	W	22.41	39.15
KAUST111	Onshore	1/22/17	Winter2016/2017	W	22.41	39.15
KAUST112	Onshore	1/23/17	Winter2016/2017	NW	22.41	39.15
KAUST123	Onshore	3/15/17	Spring2017	NW	22.41	39.15
KAUST126	Onshore	3/21/17	Spring2017	NW	22.41	39.15
KAUST127	Onshore	5/13/17	Spring2017	NW	22.41	39.15
KAUST129	Onshore	5/17/17	Spring2017	NW	22.41	39.15
KAUST130	Onshore	5/19/17	Spring2017	NW	22.41	39.15
KAUST131	Onshore	5/21/17	Spring2017	NW	22.41	39.15
KAUST150	Onshore	8/28/17	Summer2017	NW	22.41	39.15
KAUST151	Onshore	9/1/17	Summer2017	NW	22.41	39.15
KAUST152	Onshore	9/5/17	Fall2017	NW	22.41	39.15
KAUST153	Onshore	9/9/17	Fall2017	NW	22.41	39.15

KAUST157	Onshore	11/19/17	Fall2017	SW	22.41	39.15
KAUST159	Onshore	11/27/17	Fall2017	NW	22.41	39.15
Thuwal001	Offshore	2/22/16	Winter2015/2016	NE	25.28	36.8
Thuwal002	Offshore	2/27/16	Winter2015/2016	NW	25.27	36.9
Thuwal003	Offshore	3/8/16	Spring2016	NW	22.41	39.15
Thuwal004	Offshore	3/25/16	Spring2016	E	22.41	39.15
Thuwal005	Offshore	3/29/16	Spring2016	NW	27.4	34.87
Thuwal006	Offshore	3/31/16	Spring2016	NW	27.07	35.07
Thuwal009	Offshore	5/5/16	Spring2016	NW	22.41	39.15
Thuwal011	Offshore	5/11/16	Spring2016	NW	22.41	39.15
Thuwal015	Offshore	6/7/16	Summer2016	NE	22.41	39.15
Thuwal016	Offshore	7/21/16	Summer2016	NW	27.04	35.5
Thuwal017	Offshore	7/24/16	Summer2016	NW	27.04	35.5
Thuwal018	Offshore	7/24/16	Summer2016	NW	27.85	35.32
Thuwal019	Offshore	8/28/16	Summer2016	NW	27.85	35.32
Thuwal020	Offshore	9/6/16	Fall2016	NW	22.41	39.15
Thuwal021	Offshore	9/7/16	Fall2016	NW	22.41	39.15
Thuwal022	Offshore	9/21/16	Fall2016	NW	22.41	39.15
Thuwal023	Offshore	9/22/16	Fall2016	NW	19.62	39.68
Thuwal024	Offshore	9/23/16	Fall2016	NW	17.54	41.44
Thuwal025	Offshore	9/24/16	Fall2016	NW	17.66	41.09
Thuwal026	Offshore	9/25/16	Fall2016	NW	17.65	41.43
Thuwal028	Offshore	9/27/16	Fall2016	NW	18.6	40.28
Thuwal029	Offshore	9/28/16	Fall2017	NW	20.71	39.15
Thuwal030	Offshore	9/29/16	Fall2016	NW	22.41	39.15
Thuwal032	Offshore	10/5/16	Fall2016	NW	26.27	35.98
Thuwal033	Offshore	10/6/16	Fall2016	NW	26.27	35.98
Thuwal035	Offshore	10/8/16	Fall2016	NW	24.93	36.8
Thuwal037	Offshore	10/10/16	Fall2016	NW	22.41	39.15
Thuwal040	Offshore	11/8/16	Fall2016	E	26.07	35.77
Thuwal043	Offshore	11/24/16	Fall2016	NE	17.85	40.37
Thuwal044	Offshore	11/25/16	Fall2016	N	16.92	41.22
Thuwal046	Offshore	11/27/16	Fall2016	NW	17.85	40.37
Thuwal049	Offshore	11/30/16	Fall2016	SW	25.8	36.19
Thuwal050	Offshore	12/1/16	Fall2016	SW	24.2	37.1
Thuwal058	Offshore	1/20/17	Summer2016	NW	22.41	39.15
Thuwal059	Offshore	1/21/17	Winter2016/2017	NW	27.25	35.56
Thuwal060	Offshore	1/22/17	Winter2016/2017	NW	25.8	36.32
Thuwal061	Offshore	1/23/17	Winter2016/2017	NW	25.43	36.77
Thuwal062	Offshore	1/24/17	Winter2016/2017	E	23.45	37.84
Thuwal063	Offshore	1/25/17	Winter2016/2017	E	23.45	37.84
Thuwal064	Offshore	1/26/16	Summer2016	NW	22.34	38.93
Thuwal066	Offshore	3/3/17	Spring2017	NW	22.41	39.15
Thuwal067	Offshore	3/5/16	Summer2016	NW	26.64	35.83

Thuwal068	Offshore	3/7/17	Spring2017	NW	27.47	35.25
Thuwal069	Offshore	3/9/17	Spring2017	NW	25.6	36.42
Thuwal073	Offshore	3/17/17	Spring2017	NW	22.41	39.15
Thuwal078	Offshore	4/19/17	Spring2017	NW	17.6	41.48
Thuwal080	Offshore	4/23/17	Spring2017	W	22.41	39.15
Thuwal081	Offshore	7/11/17	Summer2017	NW	22.41	39.15
Thuwal082	Offshore	7/13/17	Summer2017	NW	22.41	39.15
Thuwal083	Offshore	7/15/17	Summer2017	NW	26.05	36.18
Thuwal084	Offshore	7/17/17	Summer2017	NW	18.8	40.28
TS016	Water	6/20/16	Summer2016	TS	22.31	39
TS017	Water	7/11/16	Summer2016	TS	22.31	39
TS018	Water	7/26/16	Summer2016	TS	22.31	39
TS019	Water	8/8/16	Summer2016	TS	22.31	39
TS020	Water	8/22/16	Summer2016	TS	22.31	39
TS021	Water	9/4/16	Fall2016	TS	22.31	39
TS022	Water	9/27/16	Fall2016	TS	22.31	39
TS023	Water	10/17/16	Fall2016	TS	22.31	39
TS024	Water	10/31/16	Fall2016	TS	22.31	39
TS025	Water	11/14/16	Fall2016	TS	22.31	39

Table S2. Species richness and diversity based on 16S rRNA gene sequences.

Sample	16S rRNA gene (subsampled to 5,127 reads/sample)				
	Observed OTU	Sequence Reads	Chao1 index	Shannon index	Simpson index
KAUST000	34	5130	7	1.51241577	0.7175396
KAUST001	74	5133	24	2.17291316	0.827458256
KAUST002	108	5137	43.125	2.8528788	0.929362881
KAUST003	207	5146	48	2.79985251	0.929612661
KAUST004	26	5129	19	2.36927429	0.861082206
KAUST005	25	5127	35.75	2.73048273	0.913543721
KAUST006	16	5127	17.6666667	2.30982203	0.873374574
KAUST007	42	5135	17	1.86447198	0.775051653
KAUST008	226	5136	19.2	2.71944195	0.909788167
KAUST009	110	5134	31.5	2.84190203	0.933837429
KAUST010	107	5123	24.3636364	2.57787524	0.905679012
KAUST013	18	5125	41.5	2.4791731	0.020405994
KAUST014	15	5125	33.1111111	3.09566512	0.020199917
KAUST015	48	5121	28.2	2.5219544	0.040386864
KAUST016	504	5095	31.5	2.74110017	0.234668934
KAUST017	110	5125	34.3333333	2.8003698	0
KAUST018	7	5128	31	2.81282968	0.019997959
KAUST019	149	5128	36.75	3.01401801	0.020199917
KAUST020	211	5125	29.2727273	2.72924651	0.080946181
KAUST021	285	5124	41.6	2.95982753	0.327346158
KAUST022	59	5125	13.2	2.16234981	0.871537396
KAUST023	314	5127	29.2727273	3.064728	0.311909263
KAUST024	262	5132	29.5	3.14660625	0.617301038
KAUST025	463	5141	52	2.44383141	0.127160494
KAUST026	8	5126	40.1666667	2.94008372	0.68622449
KAUST027	136	5134	51	2.91275187	0.885195445
KAUST028	421	5171	33.1666667	2.74003263	0.431511173
KAUST029	135	5147	24.9090909	2.57655263	0.040386864
KAUST030	204	5133	30	2.40348427	0.948275862
KAUST032	54	5128	25.625	3.08392096	0.894463668
KAUST034	126	5136	23.1111111	2.63558977	0.124444444
KAUST035	23	5127	30.625	2.54470255	0.154238227
KAUST036	155	5127	31.5	2.59753188	0.184929323
KAUST037	112	5136	24.5	2.79764315	0.780487805
KAUST038	458	5157	50	2.84289206	0.100609418
KAUST039	41	5128	38.1428571	3.06555471	0.753122945
KAUST040	98	5133	32.25	2.2857853	0.9168
KAUST041	355	5139	30.5	2.7702534	0.910430839

KAUST043	106	5132	16.3333333	1.60912654	0.913789263
KAUST044	380	5144	27.25	1.46852342	0.938412246
KAUST045	7	5127	32	1.45124968	0.91990032
KAUST046	201	5143	34.3333333	2.58009586	0.936903663
KAUST047	20	5129	21.25	2.61099719	0.873265157
KAUST048	532	5179	17.25	1.09091218	0.9464
KAUST052	453	5180	27	2.76240551	0.951172626
KAUST053	261	5144	22.5	2.34372939	0.890204082
KAUST054	22	5130	33	2.50369139	0.932632764
KAUST055	451	5148	34.2	2.22077036	0.930555556
KAUST056	138	5135	16	2.22980187	0.919067215
KAUST058	78	5133	11.5	1.00710049	0.898865784
KAUST059	85	5138	42.2	2.67652291	0.86612426
KAUST060	369	5144	23.1428571	2.66457682	0.946244215
KAUST061	349	5141	19.1111111	2.41172796	0.8999644
KAUST062	16	5127	30.1111111	2.30870993	0.89382716
KAUST063	78	5136	20.2	1.98496174	0.904481666
KAUST071	424	5132	25.2	2.17866527	0.913333333
KAUST077	54	5129	4	0.97616388	0.920720236
KAUST078	78	5131	3	0.28570693	0.946854352
KAUST080	322	5127	7	1.09810051	0.827755102
KAUST082	33	5132	6.5	0.97599049	0.929111531
KAUST091	108	5132	34.4285714	2.70584475	0.639841488
KAUST095	528	5164	8	0.66296575	0.581666667
KAUST098	485	5160	10.5	1.52925194	0.567708333
KAUST099	46	5132	4	0.11570538	0.898891967
KAUST100	59	5138	1	0.05741792	0.894044321
KAUST101	355	5143	1	0.05693722	0.42867036
KAUST102	107	5142	3	0.10043676	0.90446281
KAUST103	78	5130	2	0.43030386	0.88125
KAUST107	133	5151	4	0	0.879132231
KAUST108	273	5158	2	0.05646517	0.851501785
KAUST109	119	5113	2	0.05693722	0.813727435
KAUST110	125	5116	2	0.21652691	0.401249256
KAUST111	436	5034	2	0.50885878	0.892777778
KAUST112	156	5113	19.875	2.50888503	0.91768595
KAUST123	351	5081	4	0.6005015	0.8728191
KAUST126	327	5088	14	1.22619905	0.8616
KAUST127	29	5126	5.5	0.31235698	0.837409299
KAUST129	62	5122	16.5	1.71627351	0.807100592
KAUST130	166	5124	9.5	0.9546844	0.556232687
KAUST131	87	5113	2	0.10043676	0.117972154
KAUST150	106	5118	5.5	0.24493003	0.591918715
KAUST151	286	5113	2	0.28893438	0.571395537

KAUST152	319	5106	2	0.33182585	0.915345337
KAUST153	148	5129	13.6	2.01391356	0.470123457
KAUST157	240	5110	2	0.23252902	0.663957033
KAUST159	186	5125	35	1.76959283	0.041015625
Thuwal001	154	5127	18.5	2.89828677	0.932056137
Thuwal002	590	5181	17.8571429	2.44538555	0.88636841
Thuwal003	95	5137	53.8571429	3.24657845	0.953333333
Thuwal004	281	5148	46.75	2.7691205	0.92118416
Thuwal005	74	5140	21.4285714	2.61760976	0.905692081
Thuwal006	613	5196	36.0909091	3.12431544	0.94550173
Thuwal009	651	5204	15.2	2.40100913	0.877751338
Thuwal011	93	5137	27.3333333	2.22140251	0.863892699
Thuwal015	390	5144	20	1.77025204	0.71533517
Thuwal016	109	5141	24.1111111	1.84519304	0.681381274
Thuwal017	26	5129	44.2	2.71317124	0.916522491
Thuwal018	84	5129	16.75	1.93620817	0.790842872
Thuwal019	428	5133	13.5	1.51033289	0.642746914
Thuwal020	218	5136	10	0.57742061	0.221880907
Thuwal021	44	5128	61	2.76718904	0.924444444
Thuwal022	412	5175	20.5	2.62643642	0.899424717
Thuwal023	66	5138	33	2.18901835	0.833192781
Thuwal024	142	5140	21.2	2.05533314	0.804404997
Thuwal025	52	5143	19.75	2.25087871	0.860110803
Thuwal026	37	5138	16.5	1.78443811	0.690429688
Thuwal028	113	5152	38.1111111	2.53403053	0.904309715
Thuwal029	143	5146	5	1.02494384	0.553602431
Thuwal030	69	5127	12	1.35276302	0.637283951
Thuwal032	167	5082	25.3333333	1.71095076	0.679783951
Thuwal033	203	5100	32	2.37200704	0.862304688
Thuwal035	201	5077	17	1.61767962	0.701070791
Thuwal037	11	5125	24.3333333	1.79877852	0.70603538
Thuwal040	306	5111	18	1.36595951	0.551428571
Thuwal043	360	5119	5	0.70620784	0.358306926
Thuwal044	10	5126	2	0.19999966	0.095908581
Thuwal046	125	5120	15.6	2.40888956	0.877000457
Thuwal049	76	5113	2	0.20304357	0.097778722
Thuwal050	29	5115	2	0.44234015	0.270992756
Thuwal058	68	5128	4	0.96433001	0.528430226
Thuwal059	41	5118	10	0.83015179	0.472385695
Thuwal060	58	5122	4	0.89290037	0.532069971
Thuwal061	485	5022	4	0.94391354	0.497821235
Thuwal062	588	5102	4	0.68715422	0.372612847
Thuwal063	262	5113	5	0.92995136	0.474593919
Thuwal064	25	5125	8.25	1.1897644	0.523923747

Thuwal066	8	5128	2	0.13686399	0.059350271
Thuwal067	25	5126	2	0.13686399	0.059350271
Thuwal068	474	5124	2	0.13795277	0.059942608
Thuwal069	57	5122	2	0.17052986	0.078300708
Thuwal073	19	5120	3	0.24655332	0.10821281
Thuwal078	728	5122	10	1.10471359	0.45415655
Thuwal080	287	5104	2	0.20460439	0.098741319
Thuwal081	22	5121	2	0.20150888	0.096834652
Thuwal082	683	5078	4	0.91707665	0.510411951
Thuwal083	20	5123	3	0.25920334	0.133914337
Thuwal084	14	5124	5	1.17397612	0.600661626
TS016	253	5120	4	1.15879146	0.594750838
TS017	12	5126	6	1.09901731	0.634215501
TS018	63	5118	12	1.47137587	0.689919408
TS019	7	5126	4	0.9645414	0.514033499
TS020	21	5124	3	0.90025485	0.551607062
TS021	12	5124	4	0.56848191	0.25101856
TS022	24	5118	6	1.39209242	0.700740741
TS023	64	5119	8.75	1.15625821	0.54982173
TS024	47	5117	9.75	1.27536308	0.681449432
TS025	19	5117	12.3333333	0.81041146	0.366875

19

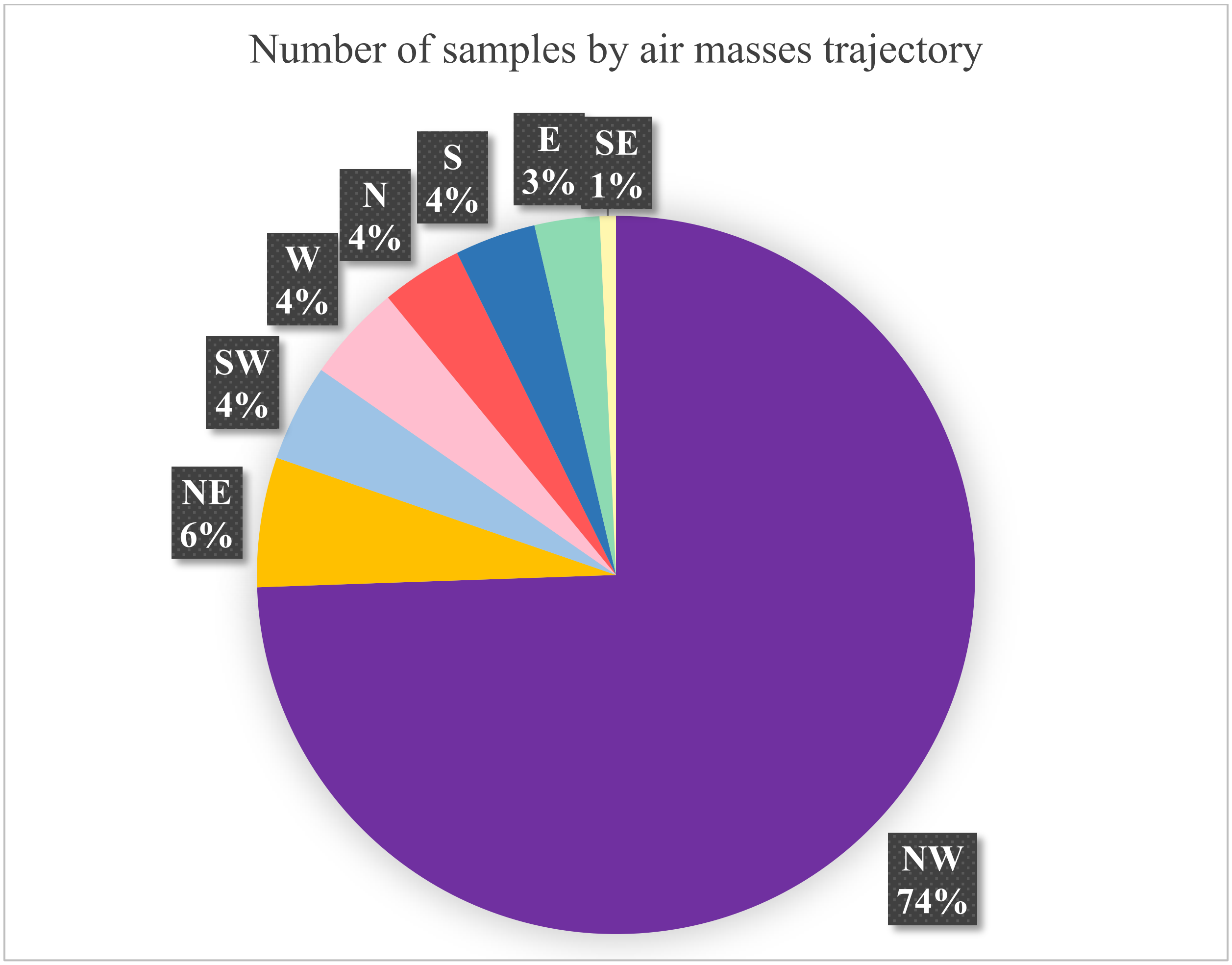

Figure S1. Pie chart showing the distribution of number of samples per air mass back trajectory.

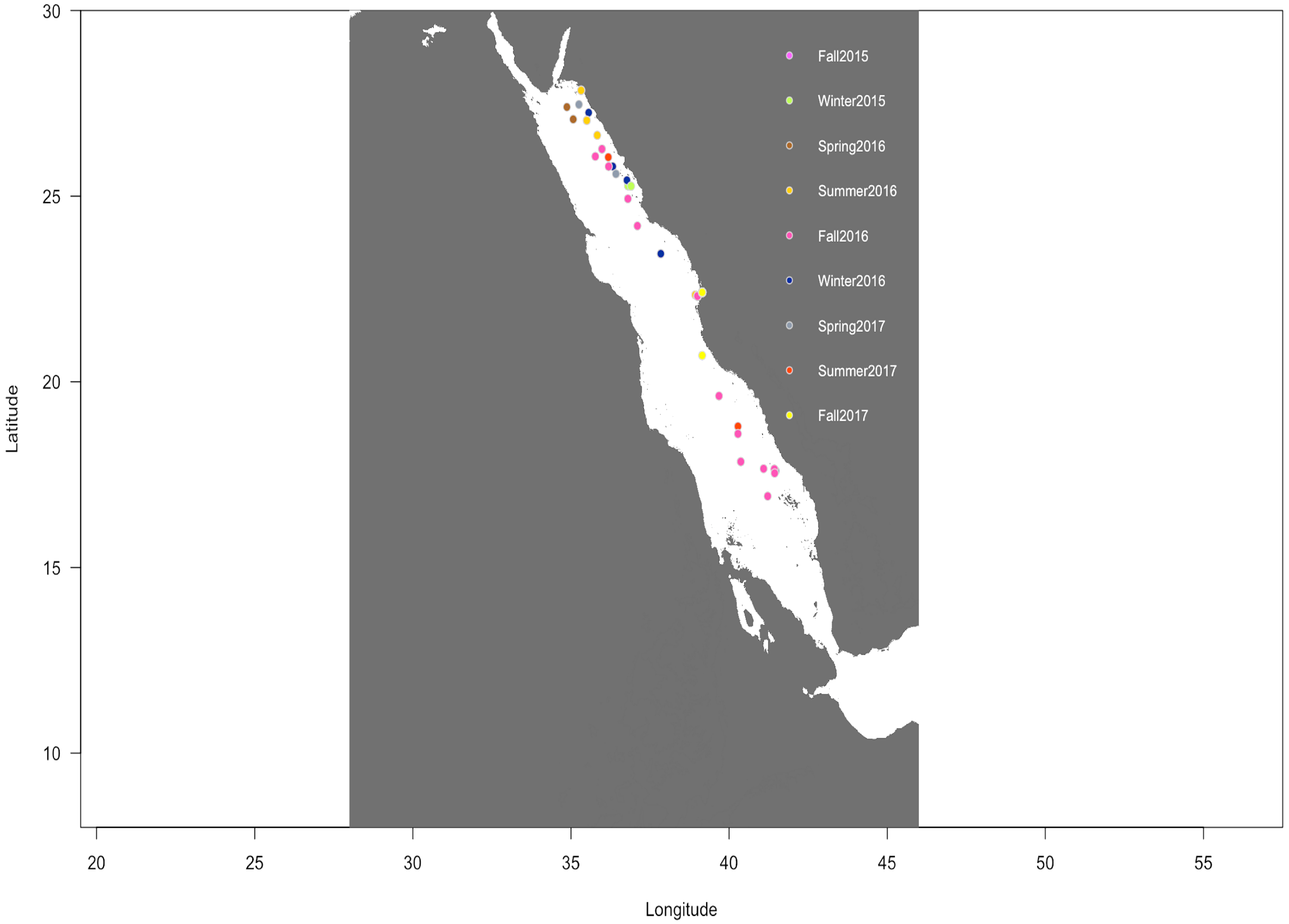

Figure S2. Map of sampling locations over the Red Sea. Colored circles show the sampling season at each location.

A

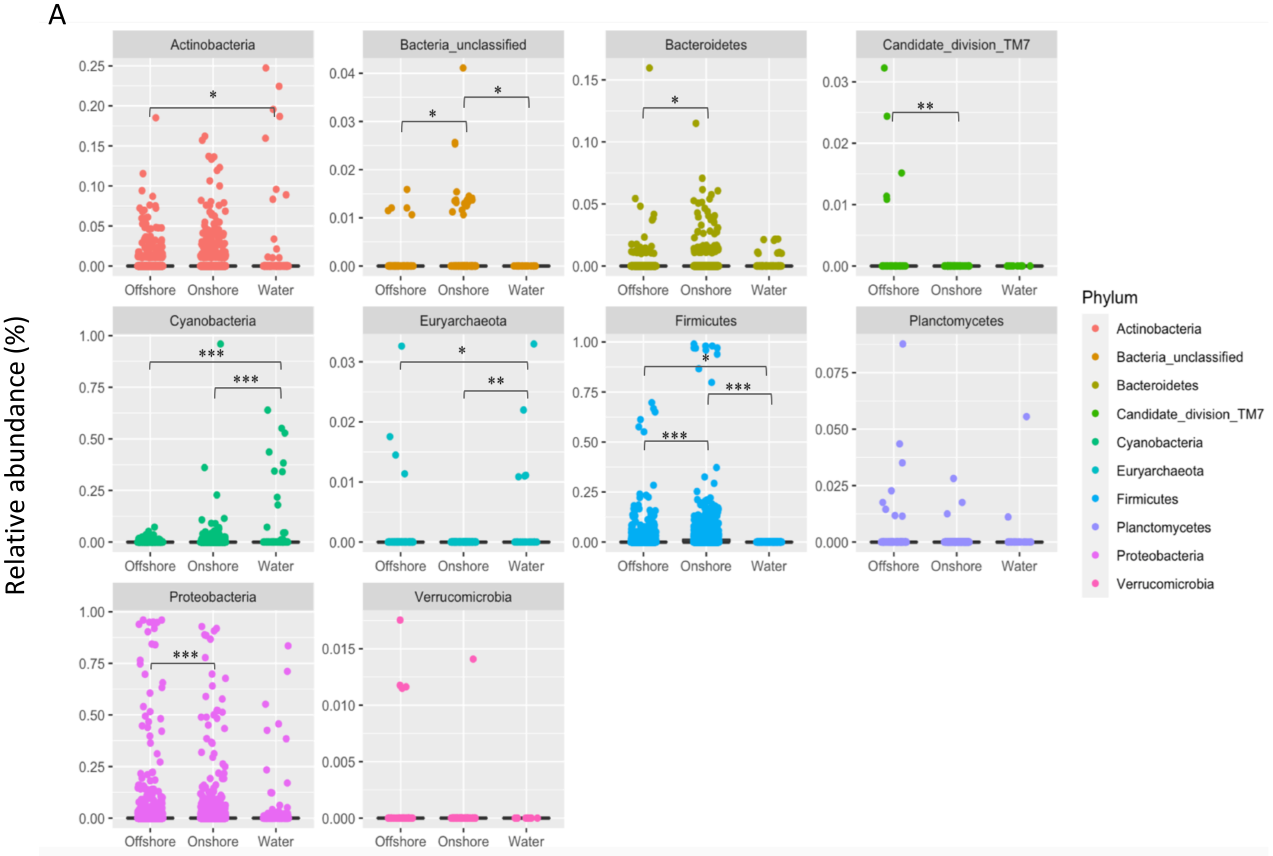

Relative abundance (%)
Actinobacteria
Bacteria_unclassified
Bacteroidetes
Candidate_division_TM7
Cyanobacteria
Euryarchaeota
Firmicutes
Planctomycetes
Proteobacteria
Verrucomicrobia
Offshore
Onshore
Water
Phylum

B

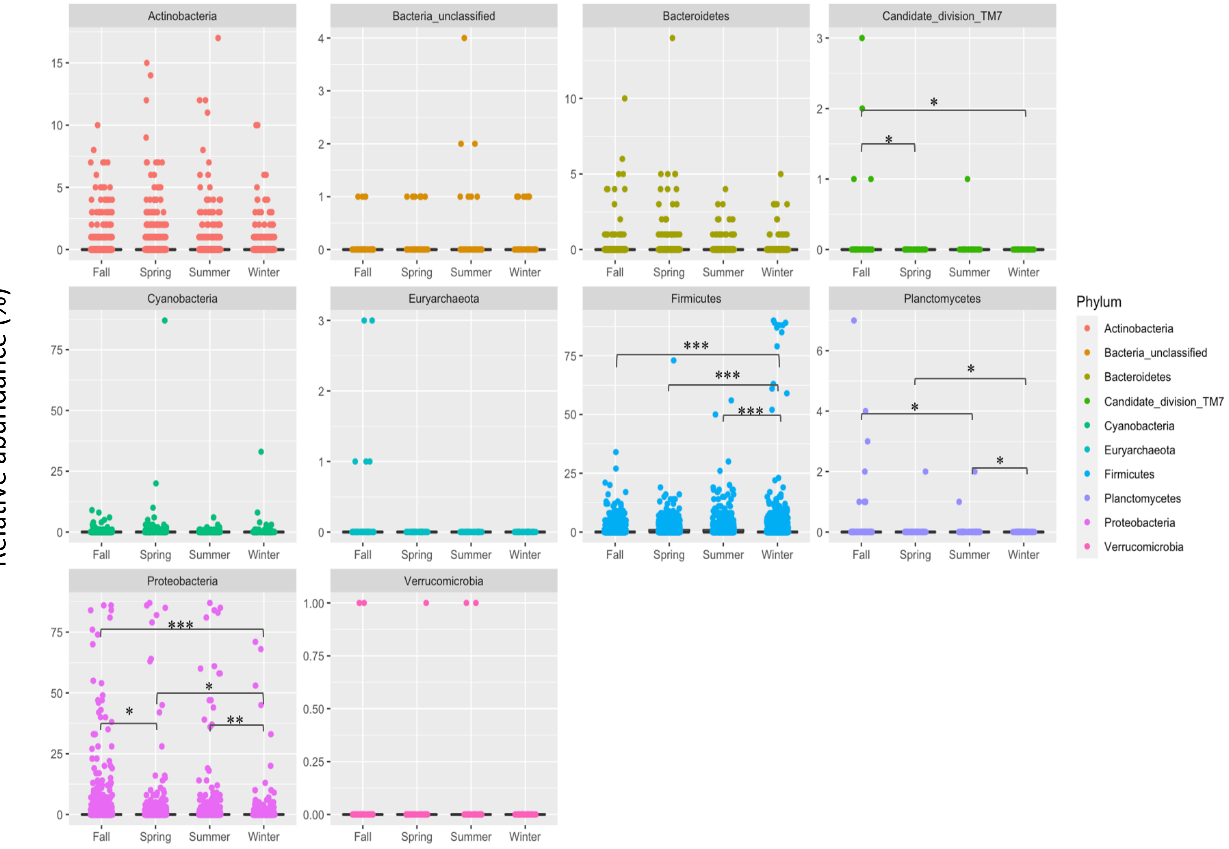

Relative abundance (%)
Actinobacteria
Bacteria_unclassified
Bacteroidetes
Candidate_division_TM7
Cyanobacteria
Euryarchaeota
Firmicutes
Planctomycetes
Proteobacteria
Verrucomicrobia
Fall
Spring
Summer
Winter
Phylum

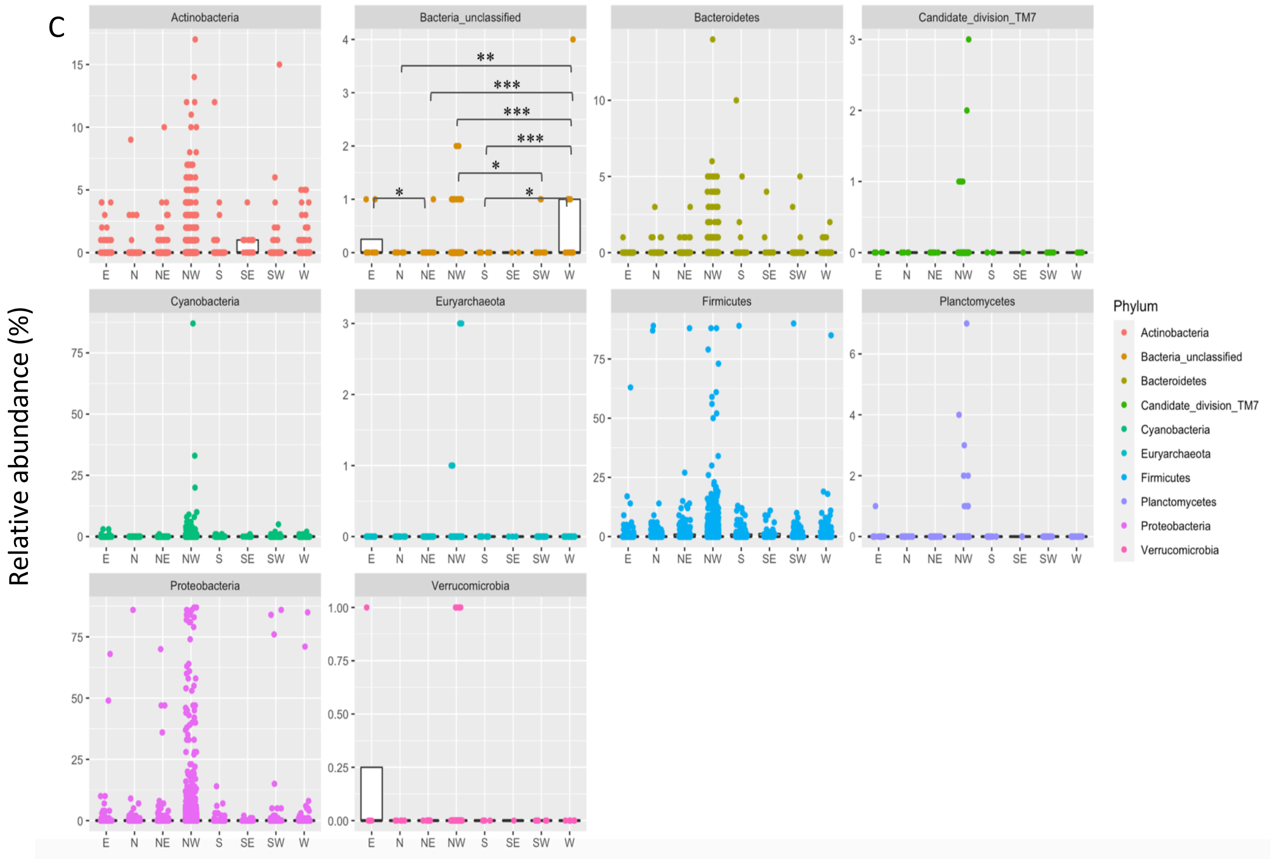

Figure S3. Differences between abundant APC phyla's relative abundances among different environmental factors. A) Sampling locations. B) Sampling seasons. C) Backward air trajectories. Significant differences were analyzed using Analysis of Variance and Bonferroni multiple comparison tests. Significant shown as *=P ≤ 0.05, **=P ≤ 0.01, and ***=P ≤ 0.001.

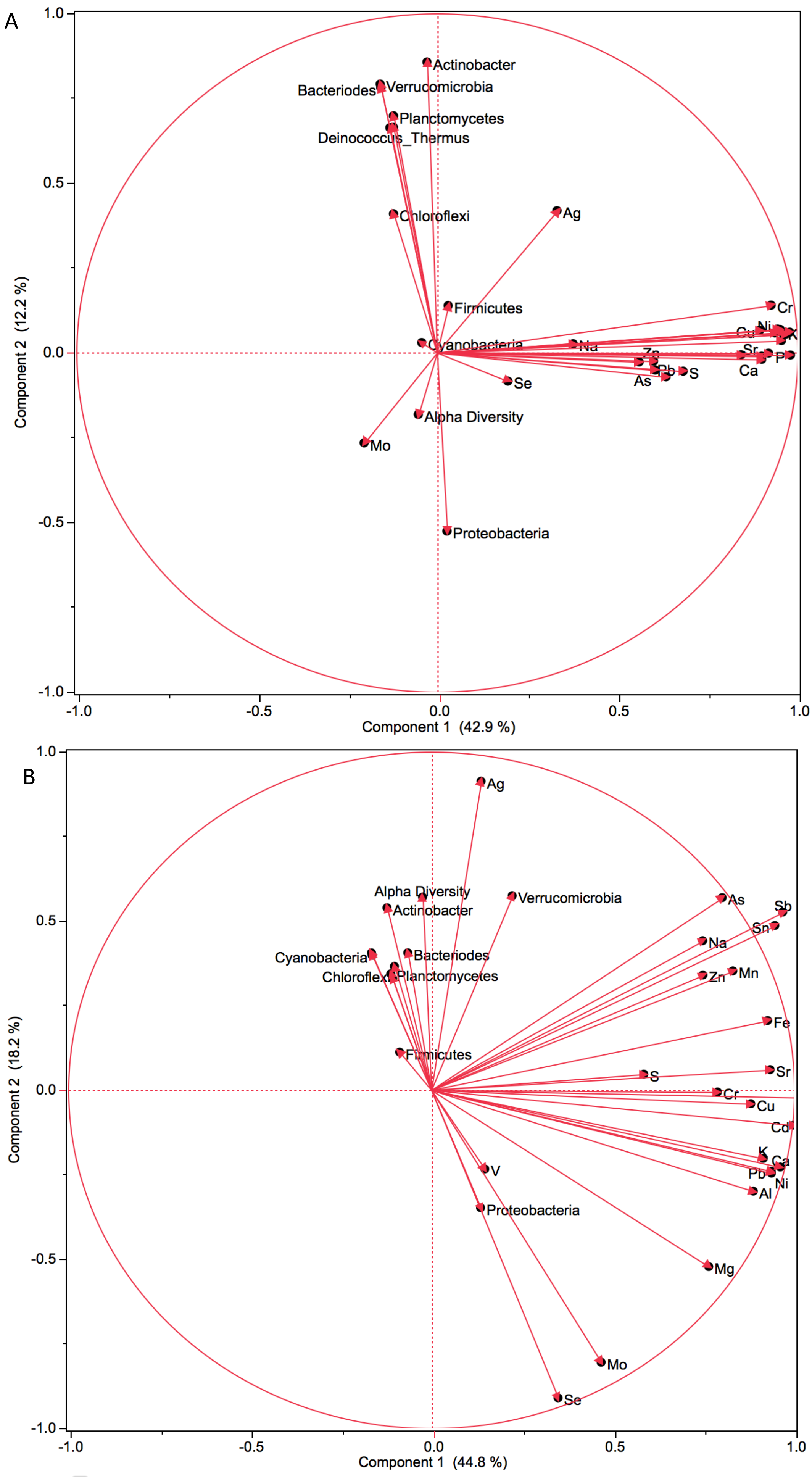
A
Actinobacter
Bacteriodes
Verrucomicrobia
Planctomycetes
Deinococcus_Thermus
Chloroflexi
Ag
Firmicutes
Cr
Ni
Cu
K
Na
Cyanobacteria
Zn
Sr
P
Pb
S
As
Ca
Se
Alpha Diversity
Mo
Proteobacteria
1.0
0.5
0.0
-0.5
-1.0
Component 2 (12.2 %)
Component 1 (42.9 %)
B
Ag
Alpha Diversity
Verrucomicrobia
Actinobacter
As
Sb
Sn
Na
Cyanobacteria
Bacteriodes
Chloroflexi
Planctomycetes
Zn
Mn
Fe
Firmicutes
S
Sr
Cr
Cu
Cd
K
Ca
Pb
Ni
Al
V
Proteobacteria
Mg
Mo
Se
1.0
0.5
0.0
-0.5
-1.0
Component 2 (18.2 %)
Component 1 (44.8 %)

Figure S4. Principal component analysis (APC) of APC as affected by trace elements concentrations based on the relative abundance of dominated phyla and alpha diversity. A) Onshore air sampling. B) Offshore air sampling. The perimeter red circle is a circle of equilibrium contribution.
